# Exercise Modulates Redox-Sensitive Small GTPase Activity in the Brain Microvasculature in a Model of Brain Metastasis Formation

**DOI:** 10.1371/journal.pone.0097033

**Published:** 2014-05-07

**Authors:** Gretchen Wolff, Jordan E. Balke, Ibolya E. Andras, Minseon Park, Michal Toborek

**Affiliations:** 1 Department of Biochemistry and Molecular Biology, University of Miami School of Medicine, Miami, Florida, United States of America; 2 Jerzy Kukuczka Academy of Physical Education, Katowice, Poland; University of Missouri-Kansas City, United States of America

## Abstract

Tumor cell extravasation into the brain requires passage through the blood-brain barrier (BBB). There is evidence that exercise can alter the oxidation status of the brain microvasculature and protect against tumor cell invasion into the brain, although the mechanisms are not well understood. In the current study, we focused on the role of microenvironment generated by exercise and metastasizing tumor cells at the levels of brain microvessels, influencing oxidative stress-mediated responses and activation of redox-sensitive small GTPases. Mature male mice were exercised for four weeks using a running wheel with the average voluntary running distance 9.0±0.3 km/day. Mice were then infused with 1.0×10^6^ D122 (murine Lewis lung carcinoma) cells into the brain microvasculature, and euthanized either 48 hours (in short-term studies) or 2–3 weeks (in long-term studies) post tumor cell administration. A significant increase in the level of reactive oxygen species was observed following 48 hours or 3 weeks of tumor cells growth, which was accompanied by a reduction in MnSOD expression in the exercised mice. Activation of the small GTPase Rho was negatively correlated with running distance in the tumor cell infused mice. Together, these data suggest that exercise may play a significant role during aggressive metastatic invasion, especially at higher intensities in pre-trained individuals.

## Introduction

While the most common cancers, such as lung, gastrointestinal, breast, and prostate cancers remain deadly diseases, there is a growing number of targeted treatments which can increase success rates [1], [2]. However, secondary metastases often develop undetected or are identified too late for successful intervention [3]. Indeed, secondary metastatic growth represents a significant source of morbidity and mortality in the US and worldwide, and the numbers could be an underestimate as postmortem autopsies identify new cases regularly [4]–[6].

It has been demonstrated that tumor and pro-oncogenic cells produce greater levels of reactive oxygen species (ROS) and have heightened levels of oxidative stress compared to normal cells [7], [8]. The increased ROS can lead to further cellular damage and progression to metastatic growth [9]. Although the exact mechanisms remain poorly understood, there is evidence that circulating factors produced by tumor cells and even tumor cell attachment can affect ROS production in surrounding cells and lead to redox imbalance [9]–[12], influencing microvascular environment. Redox-sensitive molecules such as members of the Rat sarcoma (RAS) family of small GTPases, Ras, Rho, and Rac1, have been related to increased vascular permeability that, at the level of the blood-brain barrier (BBB), may facilitate the entry of metastatic tumor cells into the brain [13]–[17].

Due to the lack of targeted therapies that can limit the dissemination of tumor cells from primary sites, it is vital to further explore the physiology of microenvironments and the pathways involved in the interactions between tumor cells and the vascular endothelium which are critical steps in tumor cell extravasation and the formation of metastases. This is particularly important for brain metastases as their detection and treatment are distinctly difficult due to the BBB that separates the brain and the rest of the body, which conveys protection and immunological privilege but provides an obstacle to treatment [18]–[20].

Exercise is one such physiological treatment with multifactorial effects on many systemic components that influence health and well-being [21]–[24]. Long-term exercise that is part of a regular schedule of activity is more adaptive and thought to produce less oxidative damage than short-term high intensity activity [25], due to increased antioxidant capacity [26]. Although associations have been made between exercise and cerebrovascular health [27], the exact mechanisms and pathways that may be activated during exercise as they relate to tumor metastasis are not completely understood [28], [29]. This is particularly true in the brain, where a unique microenvironment exists due to the presence of the BBB integrity and the lack of lymphatic circulation [30]. Therefore, the aim of this study was to evaluate the redox-regulated signaling during interactions of tumor cells with the brain microvasculature. We demonstrate that exercise can influence oxidative status of the brain microvessels and redox-sensitive signaling via small GTPases. These results suggest that exercise-redox stability in the brain endothelium may be one mechanism by which the brain is protected from metastases formation.

## Materials and Methods

### Animals and Exercise Protocol

Male C57BL/6 mice (Jackson Labs, Bar Harbor, ME), 12 weeks of age, were housed individually in plastic cages measuring 30.5×15.2×12.7 centimeters and containing a running wheel (Coulbourn Instruments, Whitehall, PA). Exercised mice had voluntary access to the running wheel and wheel revolutions were counted on an attached computer using Clocklab software (Actimetrics, Wilmette, IL). Sedentary mice had access to the same running wheel but it was locked in place. Mice were monitored for 5 weeks before tumor cell infusion and had ad libitum access to chow and water. Activity measurements were made using the final 4 weeks of exercise; the first week was used to acclimate to the wheel and solitary housing. Mice had no prior exposure to the running wheels and running was voluntary. Mice were randomly assigned to vehicle and tumor groups and analyses with running behaviors were determined posthoc. The lighting schedule was 12 hours of light followed by 12 hours of darkness, lights on at 6 AM, Eastern Standard Time. At the conclusion of the study mice were euthanized with carbon dioxide followed by decapitation.

#### Ethics Statement

All procedures, which complied with the guidelines of the American Association for Accreditation of Animal Care (AAALAC), were approved by the University of Miami Institutional Animal Care and Use Committee.

### Tumor Cell Infusion and Timeline

D122-luc, D122 cells (Lewis lung carcinoma cells tagged with luciferase, a gift from Dr. Lea Eisenbach, Weizmann Institute of Science, Rehovot, Israel) [31] were cultured in Dulbecco’s modified Eagle’s medium GlutaMax (DMEM+Glut; Invitrogen), supplemented with 10% fetal bovine serum (Invitrogen) and 1% penicillin/streptomycin (Invitrogen).

At the conclusion of the exercise regimen, mice were anesthetized with isoflurane, and the common carotid artery (CCA) was isolated along with internal (ICA) and external (ECA) carotid arteries. A 6-0 silk suture was used to ligate the end of the ECA distal to the bifurcation point while the ICA and CCA were temporarily closed with vessel clips. A small incision was made in the ECA proximal to the bifurcation point of the CCA and tubing was inserted into the CCA. After the vessel clip was removed from the ICA, 1.0×10^06^. The tubing was removed and the ECA was closed with a second suture and the remaining vessel clip removed. Blood flow returned and the surgical site was cleaned and closed.

The outcomes of exercise and tumor cell infusion were evaluated using two time points. For early effects on the induction of oxidative stress and activation of redox-regulated signaling, mice were euthanized 48 hours post tumor cell infusion (short-term studies), which coincides with transcapillary extravasation of tumor cells. The second time point was 2–3 weeks post tumor cell infusion (long-term studies), when brain metastases are fully developed. At the end of observation, the brains were extracted and microvessels isolated to measure specific changes in the microvascular endothelium.

### Microvessel Isolation

Microvessels were isolated as reported previously [32], [33]. Briefly, brains were removed and placed immediately in ice-cold PBS. Choroid plexus, meninges, cerebellum, and brain stem were removed, and brains were homogenized using isolation buffer (102 mM NaCl, 4.7 mM KCl, 2.5 mM CaCl_2_, 1.2 mM KH_2_PO_4_, 1.2 mM MgSO_4_, 15 mM HEPES, 25 mM NaHCO_3_, 10 mM glucose, 1 mM Na pyruvate with proteinase inhibitors). Then, 26% dextran (M.W. 75,000) in isolation buffer was added, and samples were centrifuged (5,800×g; 4-°C) for 20 min. The pellets were resuspended in isolation buffer and filtered through a 120 µm mesh filter paper and pelleted by centrifugation (1,500×g; 4°C) for 10 min. Samples were resuspended in Mg^2+^ lysis/wash buffer (125 mM HEPES, pH 7.5, 750 mM NaCl, 5% Igepal CA-630, 50 mM MgCl2, 5 mM EDTA and 10% glycerol with protease and phosphatase inhibitors) and incubated for 30 min at 4-°C and lysates were collected by centrifugation (14,000×g; 4-°C) for further analysis.

### DCF (2′,7′-Dichlorofluorescein) and DHE (Dihydroethidium) Fluorescence

Induction of oxidative stress was determined in homogenates of brain microvessels using 2′,7′-dichlorofluorescin diacetate (DCF-DA) and by staining of the isolated brain microvessels with dihydroethidium (DHE; both from Molecular Probes/Life Technologies, Grand Island, NY). DCF-DA is converted into the highly fluorescent 2′,7′-dichlorofluorescein (DCF) by cellular ROS, including hydrogen peroxide but excluding superoxide. DCF-DA stock solution was added to 10 µL of microvessels and PBS for a final concentration of 25 µM. Following 30 minute incubation at 37°C, fluorescence was determined at 485 nm excitation and 535 nm emission using a fluorescence plate reader. The data were expressed as specific mean fluorescence per mg protein.

DHE is membrane permeable and, in the presence of superoxide, is converted to oxyethidium, which intercalates with nucleic acids and emits a red fluorescence [34]. Freshly isolated brain microvessels were incubated with DHE (10 µM in PBS) in dark at 37°C for 90 min. After a 10 min centrifugation at 1500×g microvessels were spread on microscope slides and covered with coverslips. Images were acquired using a Nikon Eclipse Ti-U fluorescence microscope and red fluorescence intensity in the microvascular area was quantified using the NES Elements software.

### Immunoprecipitation, GTPase Activation, and Western Blotting

Protein concentrations for isolated microvessels were determined using BCA Protein Assay Kit (Thermo Scientific, Rockford, IL). Equal amounts of protein lysates (250 µg) were incubated with 40 µg of pull down assay reagents (Rac1, Rho, and Ras; Millipore Temecula, CA), which included the protein binding domain conjugated to agarose beads, at 4°C overnight with gentle rotation. The beads were collected by centrifugation (5000 rpm for 3 min) and washed with the lysis buffer described above 3 times then resuspended in 2X Laemmli reducing sample buffer. The supernatants were also collected for the measurements of the total protein concentrations and β-actin levels as a control. Equal amounts of samples were then separated on a 4–15% sodium dodecyl sulphate-polyacrylamide gel (SDS-PAGE), transferred using PVDF membranes (Bio-Rad Laboratories, Hercules, CA) and analyzed by Western Blotting. PVDF membranes were blocked with 3% bovine serum albumin (BSA) dissolved with Tris-buffered saline containing 0.1% Tween-20 (TBS-T), and incubated with the respective antibodies overnight at 4°C. Rac1 antibody was purchased from Cell Signaling (Danvers, MA); Ras, Rho, and Mn-SOD antibodies were purchased from Millipore (Temecula, CA); and β-actin antibody (recognizing both F-actin and G-actin) conjugated with peroxidase was from Sigma-Aldrich (St. Louis, MO). Antibodies for Cu/Zn-SOD, catalase and glutathione peroxidase were purchased from Abcam (Cambridge, MA). All antibodies were diluted with 3% BSA in TBS-T buffer at 1∶1000 except for the β-actin antibody which was diluted 1∶10,000. Immunoblots were analyzed by the ECL Western blot detection system (Amersham Biosciences, Piscataway, NJ).

### Statistical Analysis

Running characteristics were measured using Clocklab/Matlab software (Mathworks, Natick, MA). Western blots were quantified using ImageJ. The area under the curve (AUC) of the specific signal was corrected for the AUC of β-actin. The mean value for the treated groups was calculated according to sedentary vehicle control to determine ratios. Results were analyzed by two-way ANOVA, Student’s t test and Fisher’s LSD with significance value at p<0.05.

## Results

### Mice Display Variations in Running Distance

**Figure 1:**
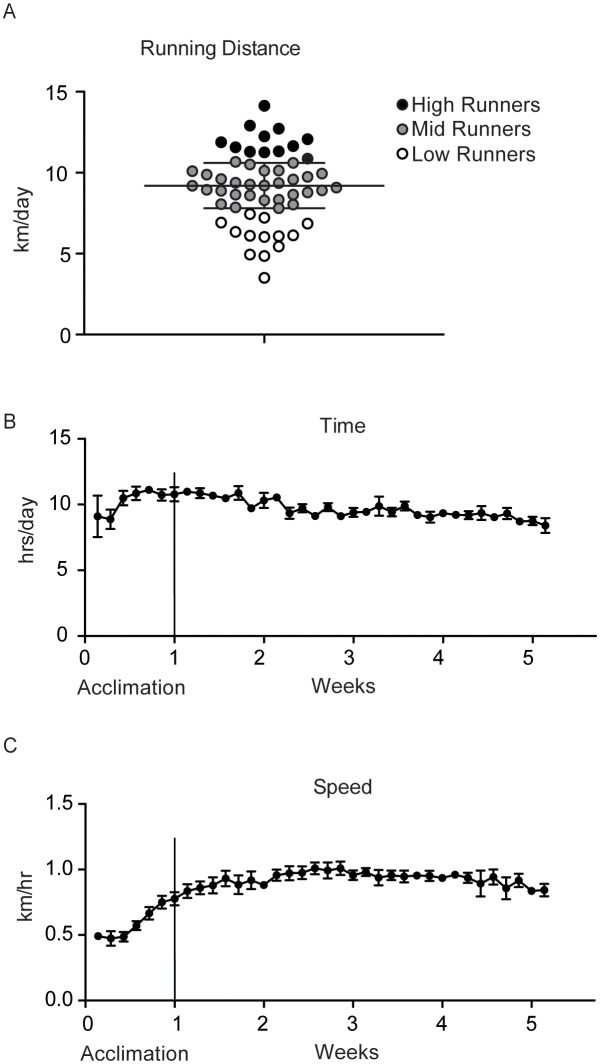
Variations in running activity in exercised mice. Mice were adapted to exercise and solitary living for one week, followed by voluntary wheel running for four weeks. (**A**) Running distance for individual mice used in the study in the last four weeks. High runners (black) run more than 10.2 km/day, mid-runners (gray), between 7.8–10.2 km/day, and low runners (white), less than 7.8 km/day. Average running distance over the four weeks of exercise was 9.0±0.3 km/day. (**B**) Time spent on running activity (hrs/day) and (**C**) speed of running. Running time and speed increased gradually during the adaptation period, then remained steady for four weeks of exercise. Average time was 9.6±0.2 hrs/day and average speed was 0.9±0.0 km/hr. Values are mean ± SEM; n = 55.

Mice who exercised for 4 weeks in a voluntary running wheel were grouped posthoc based on running distance. High runners, or the mice that ran above 10.2 km/day, mid-runners, 7.8–10.2 km/day, or low runners, below 7.8 km/day, are graphed in Figure 1A. Average running distance was 9.0±0.3 km/day. The amount of time and speed that mice were running slowly increased in the first (i.e., adaptation) week then remained fairly stable for the remaining 4 weeks (Figures 1B and 1C, respectively). Average running time over 4 weeks was 9.6±0.2 hrs/day and average speed was 0.9±0.0 km/hr.

### Superoxide Levels in Brain Microvessels of Tumor Cell-infused Mice are Inversely Correlated with Running Activity

**Figure 2:**
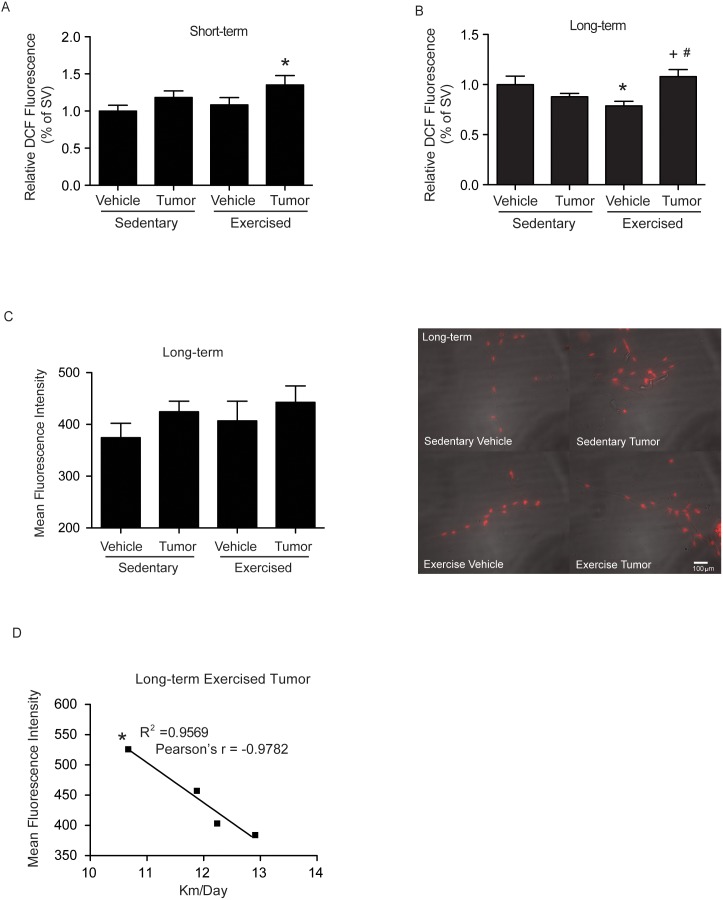
Peroxide and superoxide levels in brain microvessels of tumor cell-infused exercises and sedentary mice. Mice were exercised as in Figure 1 with sedentary mice housed in cages with locked wheels. Following exercise period, mice were infused with either 1.0×10^6^ D122 lung carcinoma cells (tumor) or cell culture media (vehicle) and euthanized 48 h (short-term studies) or 2–3 weeks (long-term studies) post tumor cell infusion. Tissue peroxides were measured by 2′,7′-dichlorofluorescein (DCF) fluorescence (**A** and **B**) and superoxide levels by dihydroethidium (DHE) fluorescence (**C**, and **D**) in freshly isolated brain microvessels. Representative DHE florescent images of freshly isolated brain microvessels visualized by staining (**C**, **right panel**). (**D**) Exercise distance negatively correlated (Pearson’s r = −0.9782) with DHE fluorescence in the tumor cell infused mice. Values are mean ± SEM, *compared to the sedentary plus vehicle group, p<0.05; +compared to sedentary plus tumor group, p<0.05; #compared with the exercised plus vehicle group, p<0.05; n = 4–15 per group.

Consistent with the notion that exercise and/or growing tumors affect tissue oxidative stress, the first series of experiments was designed to assess oxidative status of brain microvessels. Following the exercise regimen, cerebral microvessels were isolated and evaluated for oxidative stress using two separate assays. Peroxides were measured using 2′,7′-dichlorofluorescein (DCF) fluorescence. In both the short- and long-term studies, microvessels from the exercised plus tumor cell infused group showed a significant increase in peroxides (Figure 2A and 2B). In addition, the exercised plus vehicle treated group had decreased peroxides following long-term studies (Figure 2B).

DHE staining identified the presence of superoxides in brain microvessels in long-term studies. While mean fluorescence intensity was not different between the sedentary and exercised groups (Figure 2C), when running distance was compared with DHE fluorescence there was a significant negative correlation between running distance and detected superoxides when running distance was compared with DHE fluorescence intensity (Figure 2D). The high runners, in the tumor cell infused group, showed lower mean DHE mean intensity, Pearson’s r = −0.9782 (Figure 2D). Together these data suggest that following 4 weeks of voluntary wheel running, the interaction of tumor cells with brain endothelium increases the levels of ROS in brain microvessels; however, it appears that high running levels decrease superoxide production even in the presence of tumor cells. Exercise alone did not elevate superoxides or peroxides in short- or long-term studies.

### Exercise and Tumor Cell Infusion Alter Expression of Antioxidative Enzymes in Brain Microvessels

**Figure 3:**
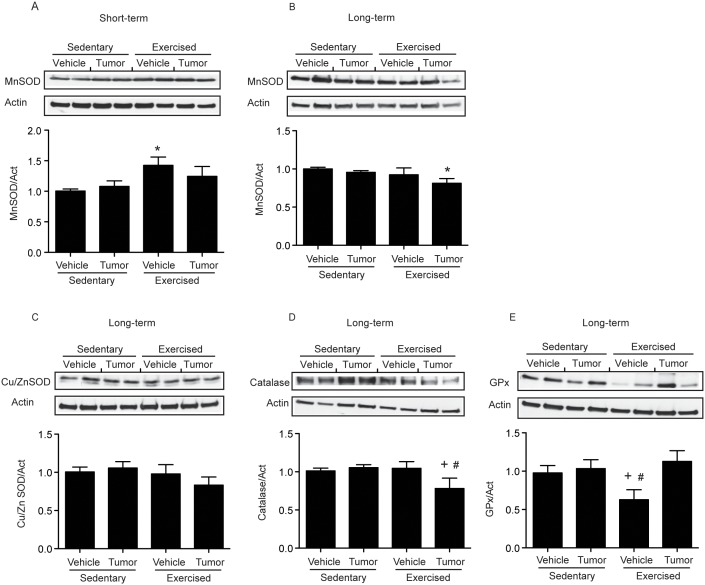
Expression of antioxidative enzymes in brain microvessels of tumor cell-infused exercised and sedentary mice. Mice were exercised and infused with tumor cells as in Figure 2, followed by evaluation of protein expression of antioxidative enzymes, MnSOD (**A**, **B**), Cu/ZnSOD (**C**), catalase (**D**), and GPx (**E**) in isolated brain microvessels by immunoblotting. The images show representative Western blots and the bar graphs reflect quantitative data from n = 6–9 per group. Values are mean ± SEM; *compared to the sedentary plus vehicle group, p<0.05; +compared to the sedentary plus tumor group, p<0.05; #compared to the exercise plus vehicle, p<0.05.

Expression of antioxidative enzymes determines tissue antioxidative capacity; therefore, protein levels of MnSOD (manganese superoxide dismutase), Cu/ZnSOD (copper/zinc superoxide dismutase), catalase, and glutathione peroxidase (GPx) were measured in exercised or sedentary mice upon infusion with tumor cells or vehicle. Protein levels of MnSOD were increased in exercised plus vehicle mice in short-term studies (Figure 3A) but decreased in the exercised plus tumor group in long-term studies (Figure 3B). In contrast, Cu/ZnSOD, showed no changes in protein levels among all studied groups, although there was a tendency towards a decrease in the exercised plus tumor group (Figure 3C). Catalase expression was markedly decreased in the exercised plus tumor group (Figure 3D). In contrast, GPx was significantly decreased in the exercised plus vehicle but not altered in the exercise plus tumor mice group (Figure 3E). Overall, these results suggest that tumor cell infusion combined with exercise reduces antioxidant capacity following tumor progression and metastasis formation.

### High Running Activity Reduces Activation of Rho GTPase in Brain Microvessels in Tumor Cell-infused Mice

**Figure 4:**
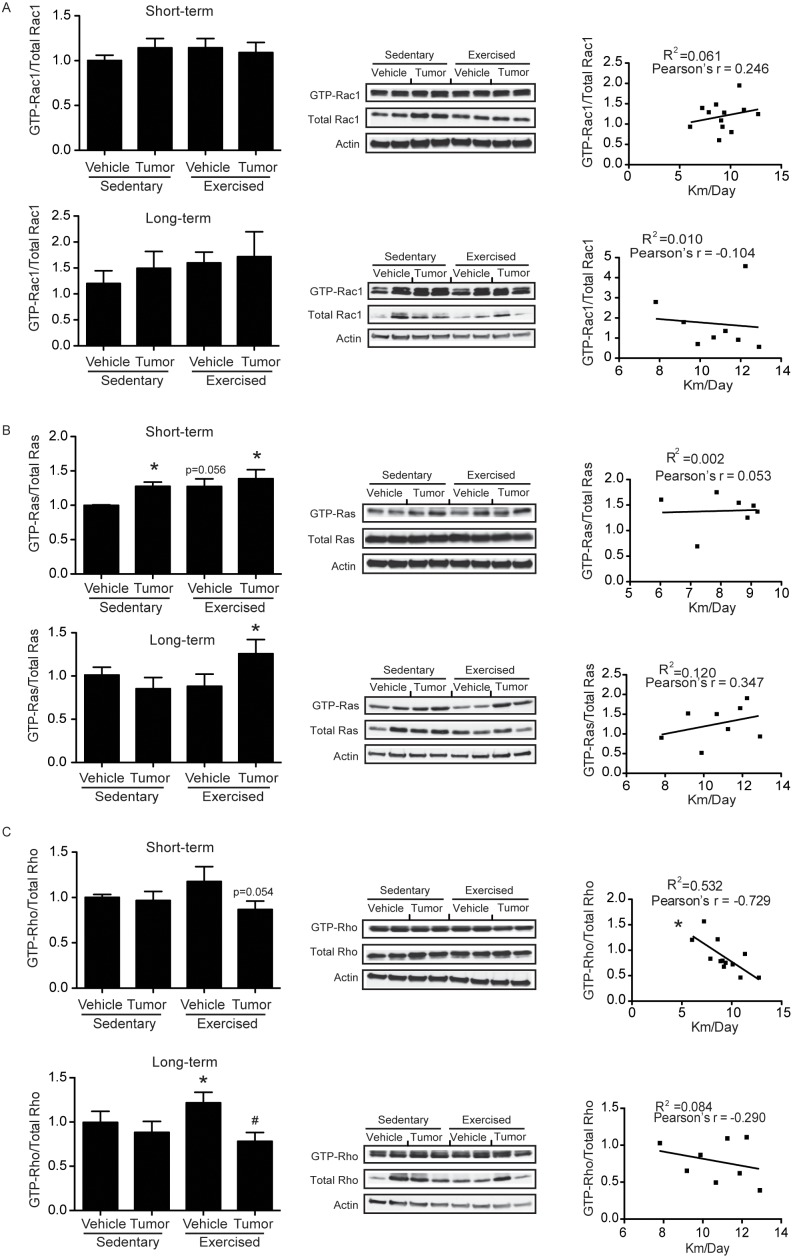
Small GTPase activity in brain microvessels of tumor cell-infused exercised and sedentary mice. Mice were exercised and infused with tumor cells as in Figure 2, followed by evaluation of active forms of Rac1 (**A**), Ras (**B**), and Rho (**C**) in isolated brain microvessels. Activation was determined using pull down assays for active (GTP-bound) GTPases, followed by immunoblotting for specific protein. In addition, a correlation between running distance and GTPase activity was determined by Pearson’s r (**A–C**). Images show representative immunoblotting from n = 8–17 and the bar graphs reflect quantitative data from these experiments. Values are mean ± SEM *compared to the sedentary plus vehicle group at p<0.05; #compared to the exercise plus vehicle group at p<0.05.

Alterations of oxidative stress are known to affect redox-related signaling. Therefore, we assessed activation of redox-sensitive small GTPases, such as Rac1, Ras, and Rho, which are involved in cell motility, proliferation, and regulation of barrier properties. Rac1 activation was not different between groups in both short- and long-term studies (Figure 4A). In short-term studies, GTP-bound Ras was significantly elevated in tumor-infused sedentary and exercised mice. A significant increase in activated Ras was also observed in the exercised plus tumor group in long-term studies (Figure 4B). However, no correlation between activated Ras and running activity was determined. In short-term studies, mean GTP bound Rho was marginally reduced in the exercised plus tumor group (Figure 4C, left). Posthoc analysis revealed that there was a significant correlation between running distance and Rho activation in this group of mice, with Pearson’s r = −0.7296 (Figure 4C, right). In long term-studies, activated Rho was elevated in the exercised plus vehicle group (Figure 4C, left); however, it was decreased in the exercised plus tumor cell cohort (Figure 4C, left).

These data indicate that redox-sensitive small GTPases in brain microvessels respond differently to stress induced by exercise, interaction of tumor cells with the brain microvessels, and/or growing brain metastases. However, it appears that exercise contributes to decreased levels of activated Rho in tumor cell infused mice, exhibiting a significant negative correlation with running distance in short-term studies.

## Discussion

Studies have shown that regular exercise and a healthy lifestyle may reduce risk for various diseases, including several types of cancer [24], [28], [35]. Mimicking such effects, exercise was introduced in the present study prior to tumor cell exposure as a model of pre-treatment. The exercise paradigm (voluntary wheel running) was chosen to model a trained individual with an active lifestyle. Mice were exercised for 5 weeks (one week of acclimation, followed by 4 weeks used for analysis) in order to avoid early changes, which may be associated with short-duration workouts. Instead, our model aimed to reproduce biological responses, which may develop in trained, regularly-exercised individuals. While mice were running for ∼9 hours per 24 hours, the activity and intensity was fully voluntary, making the model relevant to human exercise, which is also voluntary by nature. In our model, significant benefits of exercise were seen primarily in high running mice, ∼10–15 km/day, which could correspond to highly physically active humans. However, our wheel running model does not take into account other types of exercise that humans use, which contribute to overall health and disease prevention or decreased cancer progression [36].

Many studies have addressed the benefits of exercise on various cancer models; however, only few evaluated the impact of exercise on early and late stages of metastasis formation [28], [37]. Our studies specifically focused on the BBB environment, as the BBB is the most prominent of the barriers of the central nervous system (CNS), constituting one of the most impermeable interfaces in the body. The metastasis model [38] was based on infusion of a Lewis lung carcinoma cell line (mouse D122 cells) because lung tumor cells are one of the most common cancer types to home to brain tissue [3]. Our experiments focused on changes in the redox-status within the microvascular environment and the contribution of ROS following tumor cell infusion in short- and long-term studies. We have shown that in short-term studies expression of antioxidative enzymes was elevated with exercise but not in tumor groups. In addition, the levels of activated Rho GTPase were decreased in exercised tumor cell infused mice, and Rho activation was significantly negatively correlated with running distance. In long-term studies, exercise decreased superoxide levels in brain microvessels of high-running mice.

Compelling evidence demonstrates that members of the Ras superfamily of small GTPases are involved in cancer development. Many of these enzymes have conserved redox-sensitive sequences which allow for ROS meditated signaling that can be dysregulated during cancer progression [39], [40]. Indeed, Ras mutations belong to the most prevalent and well studied in the cancer field [15], [39], [41], [42]. GTPases are activated upon binding GTP and become inactive following hydrolysis of GTP to GDP. Studies have shown that small molecule oxidants can facilitate the activation of individual Ras superfamily members [39], [43], [44]. Once activated, redox-sensitive small GTPases carry out a variety of functions in the cell. For instance, Ras GTPase is involved in the regulation of cell proliferation, differentiation, and apoptosis [15], [39]. Ras GTPase is also sensitive to modulation induced by exercise. In a model of leukemia tumor growth in rats, pre-cancer exercise or exercise during cancer had no effect on the activity of antioxidant proteins, MnSOD, Cu/ZnSOD, catalase, or GPx; however Ras levels were significantly elevated in the continuously exercised rats even though the tumors were 50% of the size compared to controls [37]. In the current short- and long-term studies, we also observed an increase in activation of the redox-sensitive small GTPase Ras in brain microvessels from exercised and sedentary mice exposed to tumor cells.

Rac1 is another member of the Ras superfamily of small GTPases involved in cell-cell adhesion and cell proliferation [40]. Rac1 is both redox-sensitive and can increase ROS levels through enhanced NADPH oxidase activity and complex formation with other signaling molecules altering downstream pathways leading to cell migration [15], [39]. In the current study, Rac1 levels were not altered, indicating lack of susceptibility to exercise-mediated modification. In addition, Rac1 did not appear to be involved in the regulation of transcapillary migration of tumor cells or the growth of brain metastatic tumors.

Ras homology family member, Rho, is a redox-sensitive small GTPase that can be activated by hydrogen peroxide and is involved in actin polymerization and cytoskeleton rearrangement [39], [45], [46]. Whereas Rho is required for cell migration and adhesion, activated Rho has been associated with endothelial barrier destabilization and increased permeability during tumorigenesis [46], [47]. In the present study, we observed a decrease in Rho activation in the exercised mice infused with tumor cells in short-term studies. There was also a significant negative correlation between running distance and Rho activation, suggesting that exercise can protect microvessels from BBB instability by decreasing Rho activation.

A delicate balance exists within the tumor cell microenvironment. The same pathways, which can lead to targeted cell death and apoptosis, can be utilized to promote growth and proliferation [48]. In the case of tumor progression, mutations in several Ras related small GTPases leads to increased survival, proliferation, adhesion, and barrier permeability, favoring a metastatic phenotype [46], [47]. Therefore, keeping the balance becomes paramount and factors that can tip the scale in favor of increased barrier stability and maintenance of surrounding tissue while decreasing tumor cell survival are essential in the fight to defeat cancer.

In conclusion, exercise is a complex treatment strategy, with many physiological consequences. Associations have been shown between exercise and cancer development and outcomes, however the exact mechanisms remain elusive especially within the area of brain metastases. Further research is essential to understand the underlying signaling pathways involved and to target these for new treatment strategies, which may involve exercise in concert with other drug treatments to increase efficacy and success of current therapies.
